# Solidified glomerulosclerosis, identified using single glomerular proteomics, predicts end-stage renal disease in Chinese patients with type 2 diabetes

**DOI:** 10.1038/s41598-021-83856-z

**Published:** 2021-02-25

**Authors:** Lijun Zhao, Fang Liu, Lin Li, Junlin Zhang, Tingli Wang, Rui Zhang, Wei Zhang, Xiaoyan Yang, Xiaoxi Zeng, Yiting Wang, Yucheng Wu, Hao Yang, Shisheng Wang, Yi Zhong, Huan Xu, Shanshan Wang, Ruikun Guo, Honghong Ren, Lichuan Yang, Baihai Su, Jie Zhang, Nanwei Tong, Xin J. Zhou, Mark E. Cooper

**Affiliations:** 1 Division of Nephrology, West China Hospital of Sichuan University, No. 37, Guoxue Alley, Chengdu, Sichuan Province China; 2 Division of Pathology, West China Hospital of Sichuan University, Chengdu, Sichuan China; 3 West China Biomedical Big Data Center, West China Hospital/ West China School of Medicine of Sichuan University, Chengdu, Sichuan China; 4 Key Laboratory of Transplant Engineering and Immunology, Ministry of Health, Regenerative Medicine Research Center, West China Hospital of Sichuan University, Chengdu, Sichuan China; 5 West China-Washington Mitochondria and Metabolism Research Center, West China Hospital of Sichuan University, Chengdu, Sichuan China; 6 Division of Endocrinology, West China Hospital of Sichuan University, Chengdu, Sichuan China; 7 Department of Pathology, Baylor University Medical Center at Dallas, Dallas, TX USA; 8 Department of Diabetes, Central Clinical School, Monash University, Melbourne, Australia

**Keywords:** Diabetic nephropathy, Diabetes

## Abstract

Few histological prognostic indicators for end-stage renal disease (ESRD) have been validated in diabetic patients. This biopsy-based study aimed to identify nephropathological risk factors for ESRD in Chinese patients with type 2 diabetes. Histological features of 322 Chinese type 2 diabetic patients with biopsy-confirmed diabetic nephropathy (DN) were retrospectively analysed. Cox proportional hazards analysis was used to estimate the hazard ratio (HR) for ESRD. Single glomerular proteomics and immunohistochemistry were used to identify differentially expressed proteins and enriched pathways in glomeruli. During the median follow-up period of 24 months, 144 (45%) patients progressed to ESRD. In multivariable models, the Renal Pathology Society classification failed to predict ESRD, although the solidified glomerulosclerosis (score 1: HR 1.65, 95% confidence interval [CI] 1.04–2.60; score 2: HR 2.48, 95% CI 1.40–4.37) and extracapillary hypercellularity (HR 2.68, 95% CI 1.55–4.62) were identified as independent risk factors. Additionally, single glomerular proteomics, combined with immunohistochemistry, revealed that complement C9 and apolipoprotein E were highly expressed in solidified glomerulosclerosis. Therefore, solidified glomerulosclerosis and extracapillary hypercellularity predict diabetic ESRD in Chinese patients. Single glomerular proteomics identified solidified glomerulosclerosis as a unique pathological change that may be associated with complement overactivation and abnormal lipid metabolism.

## Introduction

The global pandemic of diabetes mellitus is possibly the largest epidemic in human history, with an estimated 463 million adults living with diabetes in 2019^1^. It is the leading cause of end-stage renal disease (ESRD) in the Western world. Likewise, diabetic nephropathy (DN) has become the major cause of ESRD in China, and develops in approximately 21.3% of patients with diabetes^2^. To date, the only widely used predictors of ESRD are clinical parameters, such as estimated glomerular filtration rate (eGFR), albuminuria, and blood pressure. However, these have limited value^3–5^. Few pathological parameters are accepted or have been validated as markers of progression to ESRD^6^.

In 2010, a pathological classification scheme for DN was developed by the Renal Pathology Society (RPS) to define the severity of diabetic kidney lesions^7^. Given the heterogeneity of kidney lesions and the complex natural history of this disease, many studies have been performed to validate the prognostic value of this scheme, but they have generated different conclusions^8–12^. Studies conducted in the USA^13^ and Japan^12^ showed that segmental sclerosis, extracapillary hypercellularity (EXHC), RPS glomerular classification, and interstitial fibrosis and tubular atrophy (IFTA) are associated with ESRD. However, the natural history and clinicopathological features of DN may differ in patients of different ethnicity. To address this issue, we analysed the histopathological features of 322 Chinese patients with type 2 diabetes (T2DM) and DN to identify pathological prognostic markers for ESRD in this population.

## Results

### Baseline features

We enrolled 322 patients, and a mean of 13 (standard deviation, 7; range, 5–39) glomeruli was obtained per biopsy. An overview of the participants is shown in Supplementary Figure S1, and their baseline clinical features are shown in Table 1. Sixty-nine percent (n = 222) were male. Median baseline eGFR and urinary protein excretion were 60.1 mL/min/1.73 m^2^ and 4.3 g/day (range, 0.2–27.0 g/day), respectively. Compared with patients who did not progress to ESRD, those who did had higher baseline proteinuria, prevalence of diabetic retinopathy, and prevalence of haematuria; and lower baseline eGFR, serum albumin concentration, and haemoglobin concentration. There were no differences in age, sex distribution, history of hypertension, duration of diabetes, body mass index, baseline fasting plasma glucose, or glycated haemoglobin (HbA1c) between the two groups. Patients who progressed to ESRD used renin–angiotensin–aldosterone system inhibitors less frequently than those who did not, although there were no significant differences in the use of other medications between the groups.

**Table 1:** Sociodemographic and clinical characteristics of the participants at the time of biopsy.

Characteristics	Total	Progressed to ESRD	Not progressed to ESRD	*P* value
	(n = 322)	(n = 144)	(n = 178)	
Age, mean (SD), years	51 (10)	51 (9)	52 (10)	0.45
Sex, Male, n (%)	222 (69)	97 (67)	125 (70)	0.58
Ethnicity				0.01
Han, n (%)	291 (90)	137 (95)	154 (87)	
Tibetan, n (%)	31 (10)	7 (5)	24 (13)	
Smoking, Never/Ex/Current, n	177/50/95	79/24/41	98/26/54	0.86
History of Hypertension, n (%)	273 (85)	128 (89)	145 (82)	0.07
BMI, mean (SD), kg/m^2^	25.5 (3.8)	25.1 (4.6)	25.7 (3.2)	0.34
SBP, mean (SD), mmHg	146 (24)	149 (23)	142 (24)	0.01
DBP, mean (SD), mmHg	87 (13)	87 (13)	86 (13)	0.19
Duration of diabetes, median (IQR), months	84 (36–132)	84 (36–132)	96 (36–132)	0.54
History of DR, n (%)	147 (46)	82 (57)	65 (37)	< 0.001
HbA1c, median (IQR), %	6.9 (5.9–8.2)	6.8 (5.7–8.2)	7.1 (6.2–8.3)	0.22
FPG, median (IQR), mg/dL	131.4 (99.0–172.8)	133.2 (95.4–181.8)	127.8 (102.6–163.8)	0.60
Hemoglobin, mean (SD), g/L	119.6 (27.6)	107.3 (20.6)	129.7 (28.5)	< 0.001
Serum albumin, mean (SD), g/L	33.7 (7.9)	29.5 (6.8)	37 (7.2)	< 0.001
eGFR, median (IQR), mL/min/1.73 m^2^	60 (43–93)	49 (34–73)	77 (52–102)	< 0.001
24-h proteinuria, median (IQR), g/d	4.3 (1.9–7.2)	6.0 (3.8–9.4)	2.7 (1.1–5.3)	< 0.001
Hematuria, n (%)	200 (62)	112 (78)	88 (49)	< 0.001
UA, mean (SD), mg/dL	6.5 (1.4)	6.5 (1.4)	6.4 (1.4)	0.59
Triglyceride, median (IQR), mg/dL	150.6 (106.3–212.6)	150.6 (106.3–212.6)	150.6 (106.3–212.6)	0.65
Cholesterol, median (IQR), mg/dL	193.9 (166.2–232.0)	201.0 (173.9–251.3)	185.6 (154.6–220.4)	< 0.001
HDL, median (IQR), mg/dL	50.3 (38.7–61.9)	54.1 (42.5–69.9)	46.4 (38.7–58.0)	< 0.001
LDL, median (IQR), mg/dL	112.1 (88.9–143.0)	116.0 (92.8–154.6)	104.4 (77.3–135.3)	< 0.001
No. of hypertensive drugs, median (IQR)	1 (0–2)	2 (1–2)	1 (0–2)	0.22
RAAS inhibitor, n (%)	260 (81)	105 (73)	155 (87)	0.01
OHA therapy, n (%)	139 (43)	63 (44)	76 (43)	0.74
Insulin therapy, n (%)	237 (74)	107 (74)	130 (73)	0.51
Statins, n (%)	191 (59)	88 (61)	103 (58)	0.57

Data are presented as means (SDs) for continuous variables with a normal distribution, as medians (25th–75th percentiles) for continuous variables without a normal distribution, and as percentages for categorical variables. BMI, body mass index; SBP, systolic blood pressure; DBP, diastolic blood pressure; MAP, mean blood pressure; DR, diabetic retinopathy; CKD, chronic kidney disease; FPG, fasting plasma glucose; BUN, blood urea nitrogen; eGFR, estimated glomerular filtration rate; UA, uric acid; HDL, high-density lipoprotein-cholesterol; LDL, low-density lipoprotein-cholesterol; RAAS, renin–angiotensin–aldosterone system inhibitor; OHA, oral hypoglycaemic agent.

### Pathological features

Pathological features of participants are shown in Supplementary Table S1. There were 17 (5%), 68 (21%), 32 (10%), 155 (48%), and 50 (16%) patients in RPS classes I, IIa, IIb, III, and IV, respectively. The percentages of patients who progressed to ESRD in classes I, IIa, IIb, III, and IV were 0%, 7%, 7%, 69%, and 17%, respectively. The percentage of patients with global glomerulosclerosis was significantly higher in those who progressed to ESRD, compared with those who did not. We next categorised global glomerulosclerosis into three patterns (Supplementary Table S2). Of these, only the prevalence of solidified glomerulosclerosis was significantly higher in patients who progressed to ESRD compared with those who did not (54% *vs*. 28%, respectively). Moreover, other glomerular lesions (mesangial expansion, Kimmelstiel–Wilson (KW) nodules, periglomerular fibrosis, capsular adhesion, EXHC, capillary microaneurysms, capsular drops, and atubular glomeruli), IFTA, tubular epithelial degeneration, and interstitial inflammation were more severe in patients who progressed to ESRD than in those who did not.

### Identification of renal prognostic factors

During a median follow-up period of 24 months, 144 (45%) patients progressed to ESRD while 17 (5%) died before entering ESRD. The results of univariable and multivariable Cox proportional hazards analyses are shown in Table 2. Univariable analysis showed that pathological parameters, including RPS glomerular classification, solidified glomerulosclerosis, obsolescent glomerulosclerosis, and KW nodules were associated with ESRD. Surprisingly, patients in class III had a higher risk of ESRD than those in class IV (hazard ratio [HR] 7.57, 95% confidence interval [CI] 3.94–14.53 and HR 6.56, 95% CI 3.13–13.72, respectively). In the fully adjusted model, only solidified glomerulosclerosis (score 1: HR 1.65, 95% CI 1.04–2.60; score 2: HR 2.48, 95% CI 1.40–4.37) and EXHC (HR 2.68, 95% CI 1.55–4.62) were significantly associated with progression to ESRD. Analysis of the incremental prognostic value of the pathological predictors showed that the model including solidified glomerulosclerosis or EXHC had a lower Akaike information criterion (AIC) than the model containing only clinical parameters (Supplementary Table S3), indicating the former model is superior. To address death as a competing outcome, we used the subdistribution hazards model (Fine and Gray method)^14^ to explore the association between clinical or pathological features and ESRD. After adjusting for the above clinical covariates and all pathological parameters, the severity of solidified glomerulosclerosis (score 1: sub-distribution hazard ratio [SHR] 1.76, 95% CI 1.11–2.81; score 2: SHR 2.39, 95% CI 1.32–4.31) and EXHC (SHR 2.74, 95% CI 1.77–4.26) remained significantly associated with ESRD in a multivariable subdistribution hazard model.

**Table 2:** Univariable and multivariable Cox proportional hazard models for progression to end-stage renal disease in patients with type 2 diabetes mellitus.

Characteristics	Univariate models	*P* value	Glomerular model	*P* value	Fully adjusted model	*P* value
	HR (95% CI)		HR (95% CI)		HR (95% CI)	
**Clinical characteristics**						
Age (+ 10 years)	0.93 (0.79–1.10)	0.41	0.96 (0.76–1.20)	0.70	0.96 (0.76–1.20)	0.72
Sex, (Female)	1.07 (0.75–1.52)	0.70	0.61 (0.38–0.99)	0.05	0.63 (0.38–1.07)	0.09
Ethnicity (Tibetan)	0.45 (0.21–0.97)	0.04	0.80 (0.35–1.84)	0.60	0.64 (0.26–1.59)	0.34
Duration of diabetes (+ 1 year)	1.01 (0.98–1.04)	0.43	1.11 (0.75–1.66)	0.60	1.28 (0.85–1.93)	0.24
Presence of DR	2.01 (1.44–2.81)	< 0.001	1.04 (0.95–1.14)	0.42	0.99 (0.9–1.10)	0.91
SBP (+ 10 mmHg)	1.12 (1.04–1.21)	0.01	0.99 (0.97–0.99)	0.04	0.99 (0.98–1.01)	0.07
Hb (+ 1 g/L)	0.97 (0.97–0.98)	< 0.001	1.02 (0.98–1.05)	0.44	1.02 (0.98–1.06)	0.26
Serum albumin (+ 1 g/L)	0.91 (0.89–0.93)	< 0.001	0.96 (0.92–0.99)	0.04	0.97 (0.93–1.01)	0.09
Cholesterol (+ 1 mg/dL)	1.15 (1.06–1.25)	< 0.01	1.03 (0.9–1.17)	0.71	1.04 (0.91–1.20)	0.54
eGFR (+ 10 mL/min/1.73 m^2^)	0.76 (0.71–0.81)	< 0.001	0.85 (0.78–0.94)	< 0.001	0.86 (0.78–0.94)	< 0.001
24-h proteinuria (+ 1 g/d)	1.10 (1.08–1.13)	< 0.001	1.02 (0.97–1.06)	0.48	1.04 (0.99–1.10)	0.13
Presence of hematuria	2.21 (1.59–3.08)	< 0.001	1.51 (0.99–2.32)	0.06	1.55 (1.01–2.39)	0.05
Usage of RAAS inhibitor	0.6 (0.42–0.87)	0.01	1.18 (0.74–1.89)	0.48	1.05 (0.64–1.71)	0.84
**Glomerular lesions**						
RPS glomerular classification						
I + IIa	1 (reference)		1 (reference)		1 (reference)	
IIb	2.38 (0.99–5.72)	0.05	0.74 (0.27–2.01)	0.55	0.63 (0.23–1.75)	0.38
III	7.57 (3.94–14.53)	< 0.001	1.92 (0.57–6.45)	0.29	2.15 (0.61–7.57)	0.24
IV	6.56 (3.13–13.72)	< 0.001	1.44 (0.52–3.97)	0.48	1.18 (0.42–3.28)	0.75
Solidified GS						
0	1 (reference)		1 (reference)		1 (reference)	
1	1.98 (1.37–2.86)	< 0.001	1.63 (1.06–2.49)	0.03	1.65 (1.04–2.60)	0.03
2 + 3	4.70 (2.98–7.42)	< 0.001	2.45 (1.39–4.29)	< 0.01	2.48 (1.40–4.37)	< 0.01
Ischemia obsolescent GS						
0	1 (reference)		1 (reference)			
1	1.68 (1.02–2.75)	0.04	1.29 (0.74–2.25)	0.38		
2	1.76 (1.07–2.9)	0.03	1.15 (0.63–2.11)	0.65		
3	1.93 (0.97–3.85)	0.06	1.44 (0.62–3.33)	0.40		
Not otherwise specified GS						
0	1 (reference)					
1	0.78 (0.45–1.33)	0.36				
2 + 3	1.05 (0.39–2.84)	0.92				
KW nodule						
0	1 (reference)		1 (reference)		1 (reference)	
1	3.67 (2.46–5.48)	< 0.001	0.64 (0.26–1.60)	0.34	0.61 (0.24–1.55)	0.30
2	4.20 (2.45–7.19)	< 0.001	0.81 (0.27–2.41)	0.71	0.77 (0.26–2.29)	0.64
3 + 4	2.74 (0.97–7.75)	0.06	0.29 (0.06–1.49)	0.14	0.47 (0.11–2.05)	0.32
Segmental sclerosis						
0	1 (reference)		1 (reference)		1 (reference)	
1	1.33 (0.95–1.87)	0.10	0.50 (0.29–0.88)	0.02	0.67 (0.44–1.02)	0.06
2 + 3 + 4	1.44 (0.66–3.12)	0.36	0.54 (0.18–1.64)	0.28	0.74 (0.29–1.87)	0.53
Periglomerular fibrosis						
0	1 (reference)		1 (reference)			
1	1.52 (1.00–2.33)	0.05	1.00 (0.61–1.64)	0.99		
2	2.05 (1.23–3.41)	0.01	1.43 (0.74–2.78)	0.29		
3 + 4	1.62 (0.57–4.65)	0.37	1.82 (0.52–6.35)	0.35		
Glomerular capsular adhesions						
0	1 (reference)		1 (reference)			
1	1.73 (1.22–2.45)	< 0.01	1.41 (0.78–2.54)	0.25		
2	2.93 (1.55–5.56)	< 0.01	1.55 (0.61–3.98)	0.36		
3 + 4	3.66 (1.15–11.68)	0.03	1.55 (0.29–8.20)	0.61		
Mesangial expansion						
1	1 (reference)		1 (reference)		1 (reference)	
2	2.36 (1.16–4.79)	0.02	1.18 (0.52–2.71)	0.69	1.12 (0.48–2.63)	0.79
3	5.85 (3.11–10.99)	< 0.001	1.12 (0.49–2.56)	0.80	1.10 (0.46–2.64)	0.83
Presence of EXHC	2.58 (1.67–3.98)	< 0.001	2.36 (1.39–3.99)	< 0.001	2.68 (1.55–4.62)	< 0.001
Presence of microaneurysm	2.78 (1.98–3.90)	< 0.001	1.24 (0.76–2.02)	0.40		
Presence of atubular glomeruli	1.69 (1.19–2.40)	< 0.01	0.85 (0.55–1.32)	0.47		
Presence of capsular drop	1.83 (1.09–3.09)	0.02	0.97 (0.52–1.83)	0.93		
Presence of fibrin cap	0.96 (0.60–1.54)	0.86				
**Interstitial lesions**						
IFTA						
0	1 (reference)				1 (reference)	
1	2.83 (0.69–11.56)	0.15			0.82 (0.09–7.45)	0.86
2	4.47 (1.09–18.39)	0.04			0.84 (0.09–7.94)	0.88
3	9.11 (2.13–39.05)	< 0.01			1.10 (0.11–11.15)	0.94
Tubular epithelial degeneration						
1	1 (reference)				1 (reference)	
2	2.87 (1.33–6.19)	0.01			1.52 (0.62–3.73)	0.36
3	5.45 (2.36–12.57)	< 0.001			1.55 (0.56–4.24)	0.40
Interstitial inflammation						
0	1 (reference)				1 (reference)	
1	6.33 (0.88–15.43)	0.07			0.67 (0.07–5.99)	0.72
2	15.95 (2.18–36.65)	0.01			0.99 (0.10–9.60)	0.96
Presence of protein cast	1.01 (0.72–1.41)	0.95				
Presence of RBC cast	1.04 (0.58–1.84)	0.90				
**Vascular lesions**						
Arteriosclerosis						
0	1 (reference)				1 (reference)	
1	1.74 (1.01–2.99)	0.05			0.64 (0.31–1.32)	0.23
2	2.07 (1.18–3.63)	0.01			0.69 (0.32–1.50)	0.35
Arteriolar hyalinosis						
0	1 (reference)				1 (reference)	
1	2.57 (1.20–5.50)	0.02			0.87 (0.32–2.40)	0.79
2	2.66 (1.29–5.50)	0.01			1.15 (0.42–3.12)	0.78

For Cox analyses, classes I and IIa were collapsed together as the reference group for RPS glomerular class. Scores of 2 and 3 were collapsed together as 2 for solidified glomerulosclerosis and not otherwise specified glomerulosclerosis. Scores of 3 and 4 were collapsed together for Kimmelstiel–Wilson nodules, glomerular capsular adhesions, and periglomerular fibrosis. Scores of 2, 3, and 4 were collapsed together for segmental sclerosis. HR, hazard ratio; DR, diabetic retinopathy; SBP, systolic blood pressure; eGFR, estimated glomerular filtration rate; RAAS: renin–angiotensin–aldosterone system; RPS, Renal Pathology Society; GS, glomerulosclerosis; KW, Kimmelstiel–Wilson; EXHC, extracapillary hypercellularity; IFTA, interstitial fibrosis and tubular atrophy.

The patients were admitted over a 15-year period, and therefore it is likely that those recruited in earlier years would have had longer follow-up periods. However, all the enrolled patients were followed up for over 1 year, and those who progressed to ESRD had a median follow-up period of 1.6 years. To determine whether the calendar years of recruitment influenced the prognostic value of pathological parameters, we further adjusted for the calendar years of recruitment in the final model, and compared the adjusted HRs in patients who were followed for at least 2 or 3 years with those who were followed for at least 1 year in the present study. The results showed that solidified glomerulosclerosis and EXHC remained independent risk factors for progression to ESRD (Supplementary Table S4).

**Figure 1:**
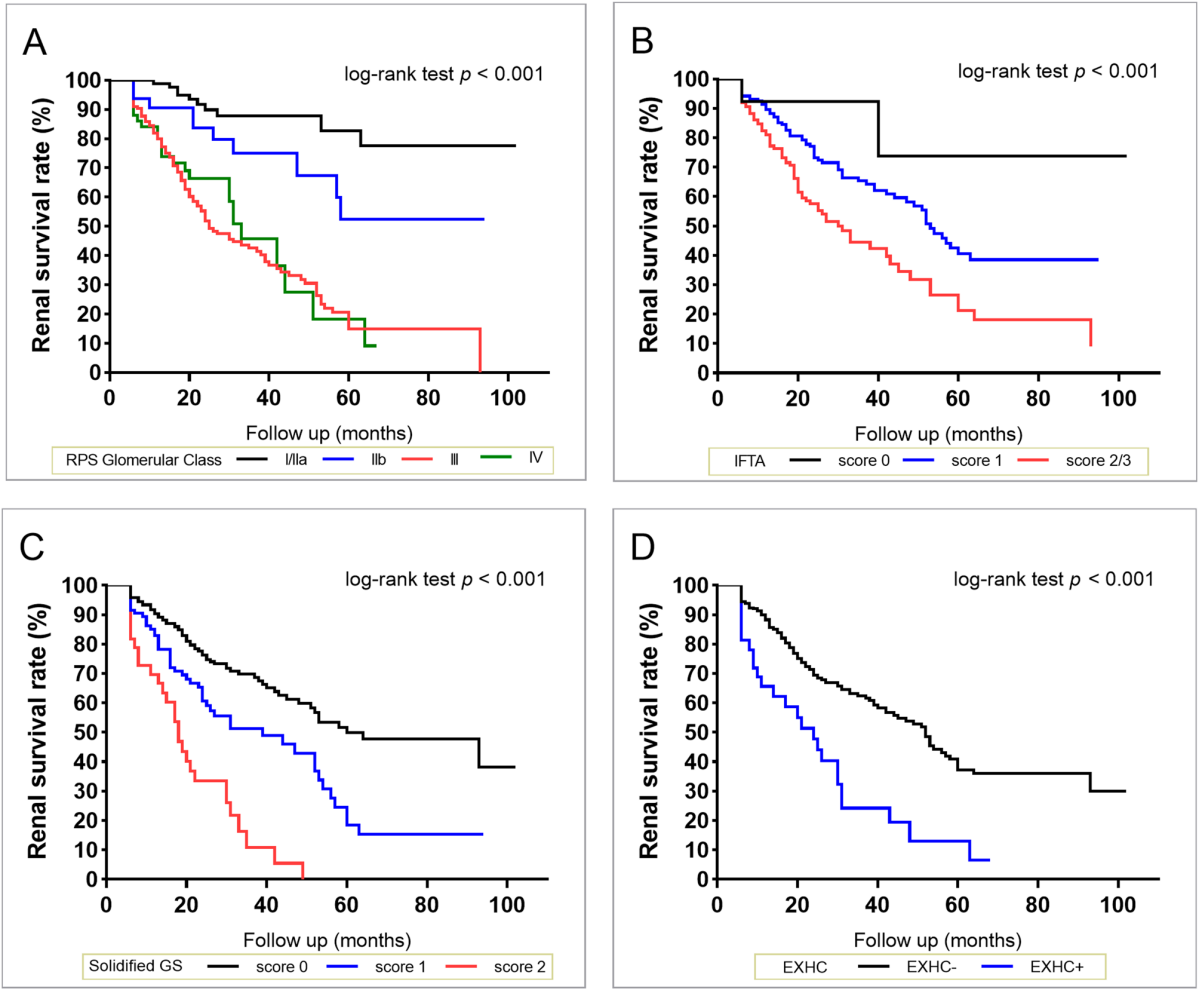
Kaplan–Meier survival curves for end-stage renal disease stratified by each pathological feature. (**A)** Renal Pathology Society glomerular classification. (**B**) Interstitial fibrosis and tubular atrophy. (**C**) Solidified glomerulosclerosis. (**D**) Extracapillary hypercellularity. RPS, Renal Pathology Society; IFTA, interstitial fibrosis and tubular atrophy; GS, glomerulosclerosis; EXHC, extracapillary hypercellularity.

Kaplan–Meier renal survival curves showed that solidified glomerulosclerosis, EXHC, RPS glomerular class, and IFTA were significant predictors of ESRD (Fig. 1). There was a graded decrease in 5-year renal survival rate with more severe solidified glomerulosclerosis. However, patients in class III had a lower 5-year renal survival rate than those in class IV (15% *vs*. 19%, respectively; Supplementary Table S5). Therefore, we analysed the proportion of global glomerulosclerosis in each sample, according to RPS glomerular classes. Of the 282 patients with global glomerulosclerosis, the proportions of solidified glomerulosclerosis in classes I, IIa, IIb, III, and IV were 0%, 10.9%, 10.9%, 24.7%, and 17.3%, respectively (*P* < 0.001) (Supplementary Figure S2). Therefore, increasing amounts of solidified glomerulosclerosis were associated with decreasing 5-year renal survival rate. Additionally, the amount of solidified glomerulosclerosis was associated with the number of KW nodules in patients with class IV pathology (*R*^2^ = 0.10, standard *β* = 0.32, *P* < 0.001).

### Clinical features of patients with solidified glomerulosclerosis

The clinical features of patients with solidified glomerulosclerosis are shown in Supplementary Table S6. Patients with solidified glomerulosclerosis had lower haemoglobin and albumin concentrations; higher serum low-density lipoprotein-cholesterol, and cholesterol concentrations; more severe proteinuria; and a higher prevalence of diabetic retinopathy than those without. Seventy-eight (61%) patients with solidified glomerulosclerosis progressed to ESRD, over a median period of 17 months.

### Proteomics of solidified glomerulosclerosis and KW lesions

**Figure 2:**
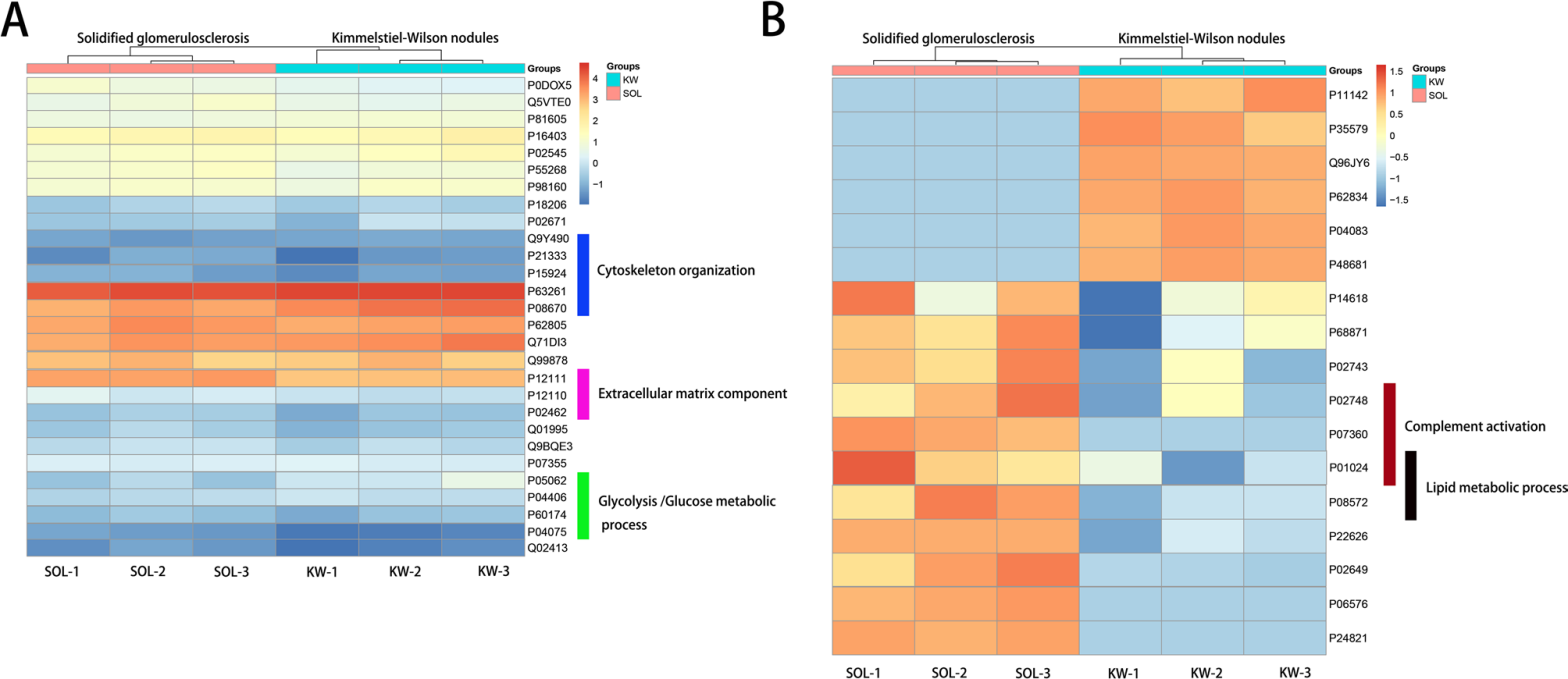
Heatmaps of the proteins identified by laser microdissection and liquid chromatography-electrospray tandem mass spectrometry of solidified glomerulosclerosis and Kimmelstiel–Wilson nodules. The heatmaps are plotted with columns representing the samples and rows representing the pathways. (**A**) Heatmap of proteins that were similarly expressed in solidified glomerulosclerosis lesions and Kimmelstiel–Wilson nodules. The blue bar represents the cytoskeleton organisation pathway, the pink bar represents the extracellular matrix components and the green bar represents glycolysis/glucose metabolic process. (**B**) Heatmap of up- and downregulated proteins in solidified glomerulosclerosis lesions and Kimmelstiel–Wilson nodules. The red bar represents the complement activation pathway and the black bar represents the lipid metabolic pathway.

Laser microdissection and liquid chromatography-electrospray tandem mass spectrometry (LC–MS/MS) identified 36 proteins that were shared and 17 that were present in significantly different amounts between solidified glomerulosclerosis and KW lesions (Supplementary Table S7). Results of the corresponding functional analysis are shown in Fig. 2. Gene Ontology (GO) and Kyoto Encyclopaedia of Genes and Genomes (KEGG) pathway analysis revealed that the proteins principally identified were extracellular matrix components (collagen alpha-1[IV] chain, collagen alpha-2[VI] chain, and collagen alpha-3[VI] chain), those involved in cytoskeleton organisation (vimentin, actin, filamin-A, and talin-1), and regulators of glycolysis (fructose-bisphosphate aldolase A/B and triosephosphate isomerase, Fig. 2A). Proteins of the complement system (complement C3/C9/C8γ) and those involved in lipid metabolism (apolipoprotein [Apo]E) were highly enriched in solidified glomerulosclerosis (Fig. 2B).

**Figure 3:**
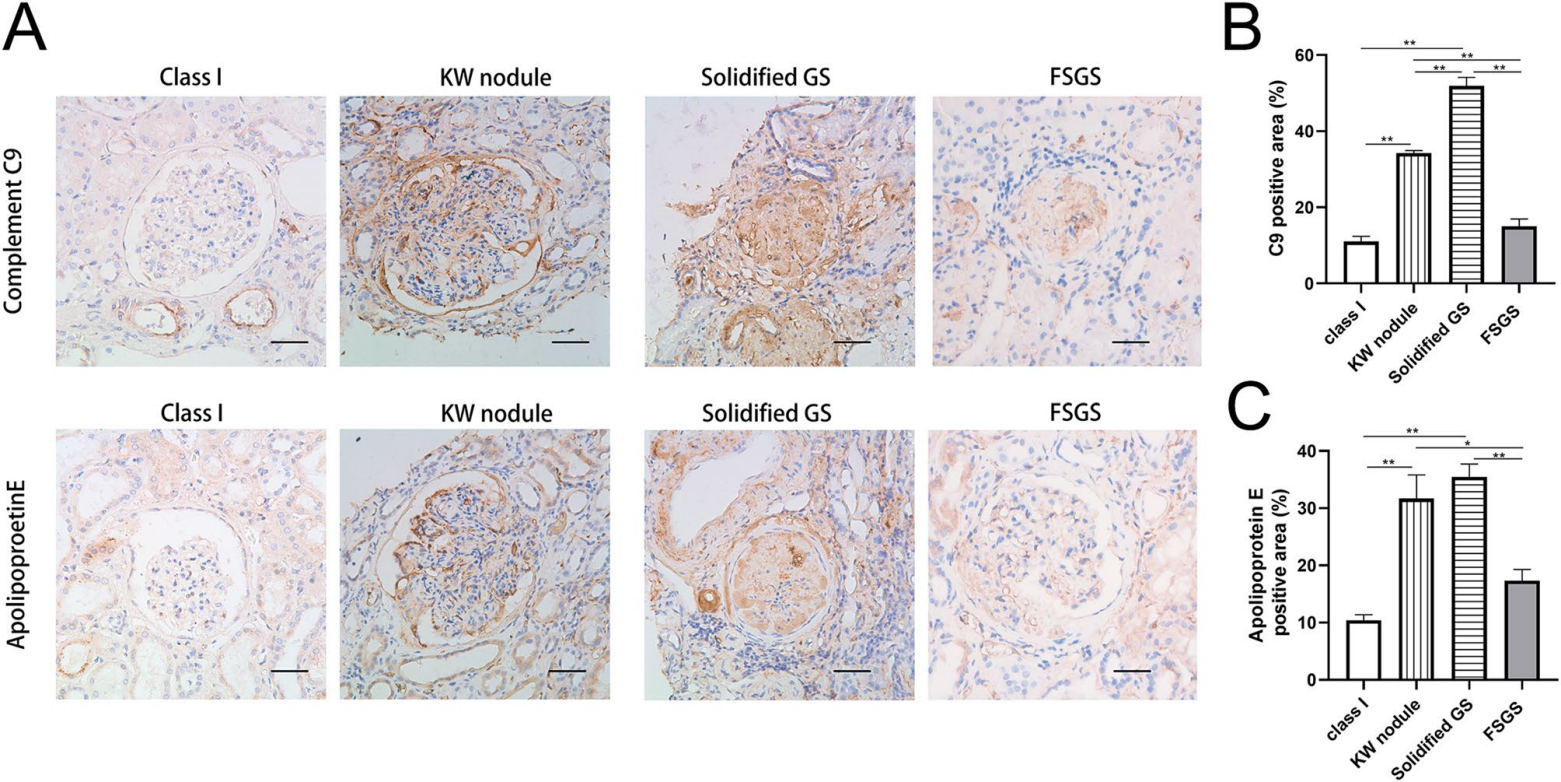
Distribution of complement C9 and apolipoprotein E in class I glomeruli in diabetic nephropathy, Kimmelstiel–Wilson (KW) nodules, solidified glomerulosclerosis (GS) and focal segmental glomerular sclerosis (FSGS). (**A**) Immunohistochemical staining for complement C9 and apolipoprotein E. (**B**) Quantification of complement C9 staining shows significant higher levels in KW nodules and solidified glomerulosclerosis (GS) than in class I glomeruli from a diabetic kidney or FSGS. (**C**) Quantification of apolipoprotein E staining shows significant higher levels in KW nodules and solidified GS than in class I glomeruli from a diabetic kidney or FSGS. Experiments were performed with samples from 10 participants for KW nodules, 10 for solidified GS, three for class I, and three for FSGS. Data are presented as mean ± SEM. * *P* < 0.05; ** *P* < 0.01. Bar = 50 µm.

To verify the differences in protein expression between solidified glomerulosclerosis and KW lesions, immunohistochemical staining for complement C9 and ApoE was performed (Fig. 3). Glomeruli from class I samples with mild changes under light microscopy were used as diabetic controls. It was reported that focal segmental glomerular sclerosis (FSGS) displays a histological pattern characteristic of glomerular solidification, which represents the predominant pattern of glomerular obsolescence^15^. Therefore, solidified glomeruli in primary FSGS were used as nondiabetic controls. Immunohistochemistry showed that complement C9 and ApoE were highly expressed in both KW nodules and solidified glomerulosclerosis compared with class I glomeruli or primary FSGS. Complement C9 was globally expressed among solidified glomeruli, and was localised in expanded mesangium and Bowman’s capsular membrane. ApoE was deposited in sclerosed areas of glomeruli or mesangial cell cytoplasm, or distributed in a granular pattern in the mesangial matrix (Fig. 3A). Moreover, consistent with the results of single glomerular proteomics, the percentage of the glomerular complement C9 and ApoE-positive area was higher in solidified glomerulosclerosis than in KW nodules (Fig. 3B,C).

## Discussion

The present study demonstrated associations between pathological features and renal outcomes in a large cohort of Chinese patients with T2DM and DN. Solidified glomerulosclerosis and EXHC are predictors of ESRD in Chinese patients with T2DM and DN, independent of clinical features. In contrast, RPS glomerular class, IFTA, interstitial inflammation, arteriosclerosis, and arteriolar hyalinosis failed to predict renal outcomes, according to multivariable models. Furthermore, single-nephron proteomic analysis showed that solidified glomerulosclerotic lesions contain structural components similar to KW nodules, and suggested the existence of increased complement activation and abnormal lipid metabolism.

Categorisation of glomerulosclerosis in diabetes was first described by Hughson et al*.* in seven patients with T2DM^15^. They found a higher prevalence of solidified glomerulosclerosis in patients with DN than in those with FSGS, IgA nephropathy, lupus nephritis, or essential hypertension. In diabetes, increasingly solidified tufts reflect marked mesangial widening, and the solidification is proposed to be associated with adaptation and segmental resistance to tuft collapse and reflow^16^, with possible concomitant mesangiolysis that is affected by a long-term diabetic state^17^. This pathophysiological process is similar to that involved in the pathogenesis of KW lesions, which have been characterised as dilated tufts with accumulation of lysed mesangial matrix^18^.

Laser capture microdissection combined with LC–MS/MS confirmed that the extracellular matrix components in solidified glomerulosclerosis and KW nodules were similar. These observations are consistent with those from previous immunohistochemical studies, which showed strong immunostaining for collagen IV, alpha-smooth muscle actin, and amorphous masses of mesangial matrix in solidified glomerulosclerosis^19^ and KW nodules^20^. Additionally, glycolytic regulators were highly enriched in both solidified glomerulosclerosis and KW nodules. Hyperglycaemia is associated with increased glucose metabolism via glycolysis ^21^. In the injured kidney, glycolysis is activated in tubular epithelium, alongside development of mitochondrial dysfunction^22^, which results in lower cellular energy production and repair capacity. Anaerobic glycolysis is also a key metabolic pathway in podocytes, which maintain the glomerular filtration barrier^23^. These findings suggest the enzymes involved in regulation of glycolysis represent potential therapeutic targets in DN.

The results of single-nephron proteomic analysis and immunohistochemistry suggested that complement factors C3, C8, and C9 are overactivated in solidified glomerulosclerosis. Accumulating evidence indicates that complement activation plays an important pathogenic role in the development and progression of DN^24^. In addition to deposition of circulating complement components in glomeruli, renal resident cells such as podocytes, mesangial cells, epithelial cells, and tubular epithelial cells have long been known to synthesise complement components^25–28^. Glomerular complement C3 deposition has been identified in both diabetic rats^29^ and human patients^30^. C3a-mediated pre-inflammatory and pre-fibrotic responses in diabetic rats aggravate renal injury, while C3a receptor antagonists improve renal function in streptozotocin-induced diabetic rats ^31^. Complement C9 binds C8 in the C5b-8 complex, and forms the C5b-9 membrane attack complex (MAC). Increased tissue deposition of the C5b-9 MAC activates intracellular signalling pathways, which promote release of proinflammatory cytokines and growth factors^32^. Immunoelectron and immunofluorescence microscopic studies of diabetic renal biopsy specimens showed a direct correlation between the magnitude of mesangial expansion and the level of mesangial MAC deposition, using antibodies directed against the C9 component^33^. While C7 was not detected in either solidified glomeruli or KW lesions in the present study, this may have been related to protein loss in formalin-fixed, paraffin-embedded tissue^34^. Immunohistochemical staining confirmed increased staining of C7 in early DN kidneys compared with nondiabetic controls^35^. Furthermore, urinary concentrations of C3, C7, and C5b-9 correlate significantly with urinary protein excretion and eGFR in patients with diabetes^36,37^. However, it is less likely that nonspecific trapping of complement components in solidified glomeruli associated with DN occurs, because solidified glomeruli in FSGS do not show significant complement deposition, as in the present study. Therefore, it is reasonable to speculate that inhibition of the complement system may represent a means of preventing glomerular solidification.

The present data showed that ApoE, a key regulator of lipid and lipoprotein homeostasis, was present at significantly higher concentrations in solidified glomerulosclerosis. Similar findings were previously reported by Takemura et al.^38^ and Sao et al.^39^. ApoE is a ligand for low-density lipoprotein receptor family members, which mediate clearance of ApoB-containing atherogenic lipoproteins. Immunocytochemical staining of renal biopsy specimens featuring various types of glomerulonephritis showed deposition of ApoE and ApoB in mesangium, sclerosed areas of glomeruli, and glomerular cells. This deposition was primarily receptor-mediated and was associated with the severity of mesangial expansion, glomerular sclerosis, and proteinuria^38–40^. Cultured podocytes showed a high affinity for uptake of ApoE-containing lipoproteins. Plasma accumulation of ApoE-rich lipoproteins in nephrosis resulted in exposure of podocytes to relatively high concentrations of these lipoproteins. Elevated uptake of ApoE-containing lipoproteins may contribute to glomerular lipid accumulation and sclerosis^40^. Because ectopic lipid deposition in hyalinosis lesions has been observed in solidified glomerulosclerosis in diabetic patients^15,41^, ApoE-positive lipid droplets in mesangium may exert an effect similar to that of modified low-density lipoprotein on mesangial cells^42^, which results in glomerular injury, followed by sclerosis. Because patients with solidified glomerulosclerosis had higher cholesterol level and glomerular deposition of ApoE than those without solidified glomerulosclerosis, it is likely that glomerular deposition of apolipoproteins partly reflects higher circulating concentrations of these substances.

The process of ischaemic obsolescence has long been believed to be the consequence of decreased glomerular perfusion, resulting from narrowing of the intrarenal vasculature or normal aging^16^. Mixed patterns of solidified glomerulosclerosis and ischaemic obsolescent glomerulosclerosis were observed in 111 (35%) biopsy specimens in the present study. In the corresponding patients, glomerular loss may have resulted from combination of glomerular disease and ischaemia, secondary to hypertension-induced vascular changes^15^.

The use of EXHC as a prognostic indicator of ESRD in patients with diabetes was first described by Mottl et al.^13^. The origin of EXHC, whether from parietal epithelial cells or podocytes, remains unclear. The proliferation of parietal epithelial cells in EXHC was reported to be involved in progression of glomerulosclerosis^43^. However, it is difficult to make a clear distinction between EXHC and a true crescent. Diabetic crescent formation may result from glomerular basement membrane rupture because of the presence of a hyaline cap, which develops at sites of mesangiolysis and capillary aneurysm formation^13,44^. Because the patients in the present cohort were all antineutrophil cytoplasmic antibody and antiglomerular basement membrane antibody-negative and did not have coexisting immune complex disease, the EXHC cannot be explained by these aetiologies. Therefore, in diabetic patients, in the absence of a secondary aetiology, it is likely that EXHC did not represent an inflammatory change, rather a DN-induced glomerular injury related to a diabetic crescent^45^.

The pathological parameters used in the RPS classification were not associated with renal outcomes in the present study, which is consistent with findings of a previous study^13^. KW nodules have been reported to be independent risk factor for poor renal prognosis^46,47^. However, solidified glomerulosclerosis, rather than KW nodules, was identified to be an independent risk factor for ESRD in the present study. Solidified glomerulosclerosis existed in patients in classes IIa–IV, whereas by definition, KW nodules were only present in classes III and IV. Therefore, for patients in the early stages of DN, solidified glomerulosclerosis may be a more useful predictor of clinical outcome. Furthermore, early intervention may be more beneficial in patients with smaller amounts of solidified glomerulosclerosis.

Our results showed that diabetes duration, HbA1c, and fasting plasma glucose were similar between patients who progressed to ESRD and those who did not, which was consistent with previous studies^48,49^. In a prospective analysis of patients with T2DM, the coefficient of HbA1c variation, not the mean of serially measured fasting plasma glucose, was predictive of the development of renal complications and diabetes-related outcomes^50^. Wide variance of HbA1c reflects a more complicated clinical course, suboptimal use of medications, and/or self-management^50^. The absence of an association between diabetes duration and renal outcome may be explained by the inability to establish the time of onset of T2DM with certainty. Many individuals have undiagnosed diabetes, and impaired glucose tolerance for extended periods leads to inaccurate assessment of diabetes duration^51^.

The present study had several limitations. Renal biopsies were performed based on clinical indications, rather than a research protocol. Therefore, there may have been intrinsic selection bias. The patients were enrolled in tertiary referral hospitals with a clinical requirement for renal biopsy and did not represent the typical findings of nephropathy in type 2 diabetic patients with renal disease. Another potential limitation was that this was a single-centre retrospective study in which only patients with T2DM were enrolled. In the future, the pathological risk factors for ESRD should also be evaluated in prospective studies, in type 1 diabetes patients, and across multiple centres. Lastly, the minimum number of glomeruli per patient for analysis was set at five in the present study, which may have influenced glomerular disease classification.

Our findings show that solidified glomerulosclerosis, as a novel pathological parameter, and EXHC, are independent predictors of ESRD in Chinese patients with T2DM. Prospective studies in larger cohorts are necessary to further validate the prognostic utility of these pathological findings.

## Methods

### Patient selection and study design

We conducted a retrospective study of patients with T2DM and DN who had undergone percutaneous renal biopsy between 2003 and 2018 at the West China Hospital of Sichuan University. Renal biopsy was performed in T2DM patients with renal injury who lacked absolute contraindications^8,52^. The indication for renal biopsy was diabetes with persistent albuminuria or renal dysfunction, particularly with sudden onset overt proteinuria or haematuria^53^. Absolute contraindications to renal biopsy have historically included uncontrolled severe hypertension, inability to cooperate with biopsy, solitary kidney, and uncontrollable bleeding diathesis^54^. Diagnosis of T2DM was made according to American Diabetes Association criteria^55^. DN was diagnosed by at least two renal pathologists and/or nephrologists using the RPS classification^7^. Adult patients with T2DM and biopsy-confirmed DN who were followed up at our hospital for at least 1 year were eligible for the study. Patients who progressed to ESRD within 1 year of renal biopsy were also included in the study. Exclusion criteria were the presence of coexisting nondiabetic renal diseases, such as IgA nephropathy; systemic diseases, such as antineutrophil cytoplasmic antibody-associated vasculitis, anti-glomerular basement membrane disease, or lupus nephritis; progression to ESRD prior to renal biopsy; lack of follow-up data; and presence of fewer than five glomeruli per biopsy specimen (Supplementary Figure S3). Ultimately, 322 patients with pure DN were enrolled. All patients provided written informed consent and the study was approved by the Institutional Review Board of the West China Hospital of Sichuan University [Approval number was 2003 (1)].

### Clinical and laboratory data

All baseline demographic and clinical data were collected at the time of renal biopsy. The eGFR was calculated using the creatinine-based Chronic Kidney Disease Epidemiology Collaboration Eq.^49^. Smoking status was determined at the time of biopsy. Diabetic retinopathy was defined as the existence of microaneurysms, retinal dot or blot haemorrhages, or neovascularisation in the retina. Haematuria was defined as the presence of more than five erythrocytes per high-power field in at least two of three consecutive urine tests, in the absence of urinary tract infection, malignancy or lithiasis^12,49^. The use of renin–angiotensin–aldosterone system inhibitors, glucose-lowering agents and statins by the patient for more than half of the follow-up period was defined as treatment. Detailed follow-up data were recorded over a mean of three visits per patient per year.

### Histopathological findings

**Figure 4:**
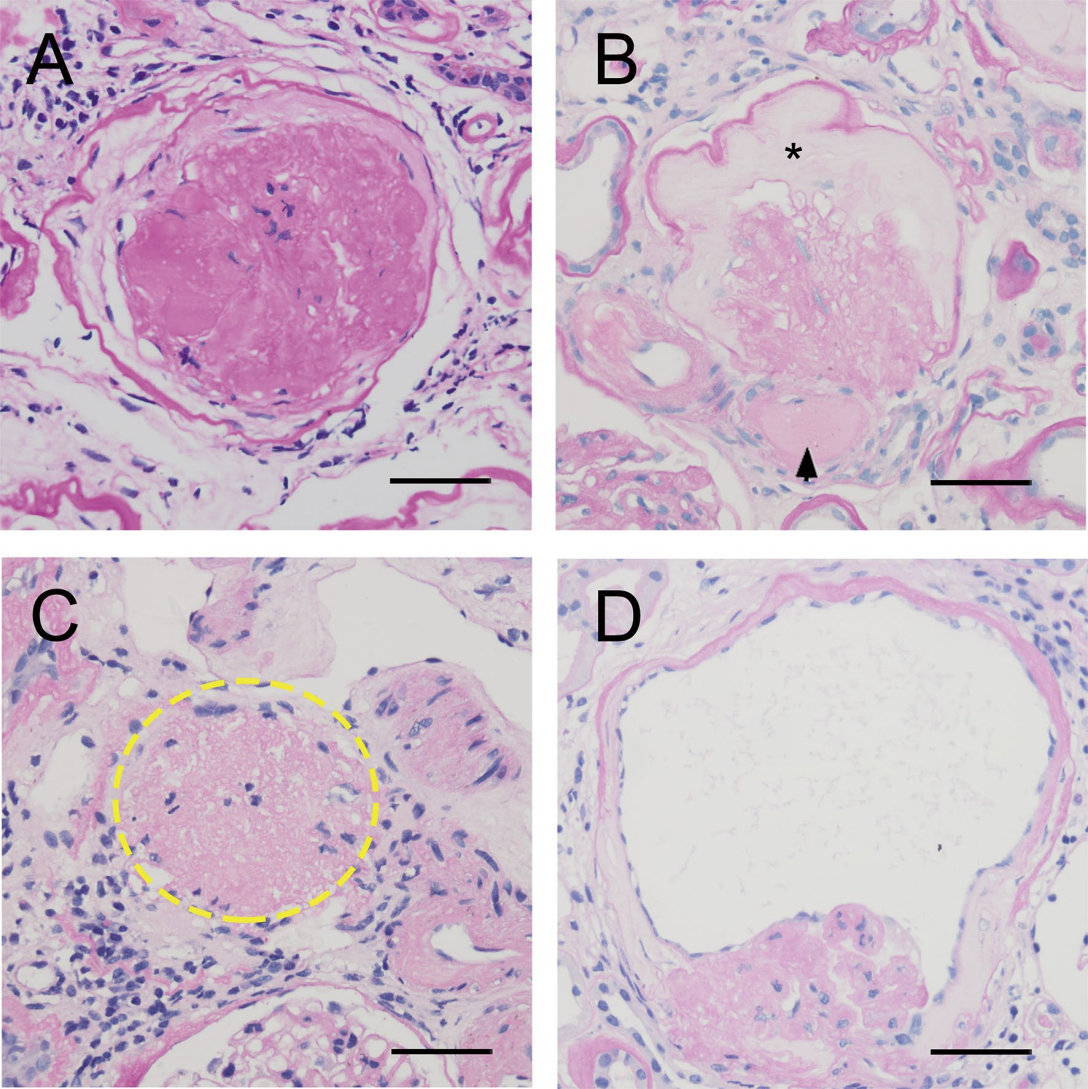
Light photomicrographs of the different patterns of global glomerulosclerosis in diabetic nephropathy. (**A**) Solidified glomerulosclerosis is characterised by expansion of the mesangial matrix of the glomerular tuft, which fills Bowman’s capsule (periodic acid-Schiff [PAS], × 400). (**B**) Obsolescent glomerulosclerosis is characterised by wrinkling of the glomerular basement membrane, with subsequent glomerular tuft retraction toward the vascular pole, and gradual accumulation of collagenous connective tissue (stars), beginning at the vascular pole adjacent to the glomerular stalk. Severe arteriolar hyalinosis (arrowhead) can also be observed (PAS, × 400). (**C**) An example of not otherwise specified glomerulosclerosis, showing disappearing glomerulosclerosis (dashed line), and absence or partial disappearance of Bowman’s capsule, such that there is a continuum between the sclerotic glomerulus and the fibrotic interstitium (PAS, × 400). (**D**) Another example of not otherwise specified glomerulosclerosis, showing a shrunken glomerulus surrounded by inflamed interstitium (PAS, × 400). Bar = 50 µm.

Renal biopsy samples were prepared for light microscopy, immunofluorescence and electron microscopy using standard procedures at West China Hospital. For light microscopic examination, renal specimens were stained with haematoxylin and eosin, periodic acid-Schiff, Masson’s trichrome and periodic acid-Schiff silver methenamine. Immunofluorescence staining was performed for IgG, IgM, IgA, C1q, C3, albumin, fibrinogen, and kappa and lambda light chains. The pathological features evaluated using light microscopy are listed in Supplementary Table S1. In addition to the RPS DN classification^7^, numerous common pathological lesions were evaluated, including EXHC (Supplementary Figure S4), segmental sclerosis, KW lesion, periglomerular fibrosis and glomerular capsular adhesion, mesangial expansion, capillary microaneurysm, capsular drop, fibrin cap and atubular glomerulus^13,16,56^. Atubular glomerulus was defined as a glomerulus attached to a short atrophic segment of proximal tubule, with a tip lesion at the glomerulotubular junction^56^. Global glomerulosclerosis was classified as solidified, ischemic obsolescent or not otherwise specified. They were defined as follows:

1. Solidified glomerulosclerosis was defined as glomeruli in which the entire tuft was solidified with increasing accumulation of mesangial matrix that expands the glomerular tuft, with the absence of collagenous changes in the capsular space (Fig. 4A)^15,16^.
2. Ischemic obsolescent glomerulosclerosis was characterized by a retracted glomerular tuft and surrounded by a hypocellular homogeneous collagen matrix beginning at the vascular pole adjacent to the glomerular stalk (Fig. 4B)^16,19^.
3. Not otherwise specified glomerulosclerosis included disappearing glomerulosclerosis and non-specific defined glomerulosclerosis. Disappearing glomerulosclerosis was defined by the absence or partial disappearance of Bowman’s capsule (Fig. 4C)^16^. Sclerotic glomeruli that could not be defined as solidified, ischemic obsolescent, or disappearing were assigned to the not otherwise specified glomerulosclerosis category (Fig. 4D).

IFTA, interstitial inflammation, arteriosclerosis and arteriolar hyalinosis were assessed and scored according to the RPS DN classification^7^. Epithelial tubular degeneration^57^, protein casts and red blood cell casts were further reviewed. Renal specimens were examined by two nephropathologists (L.L. and H.X.) and any scoring discrepancies were resolved by discussion. The inter-observer agreement for the scoring of global glomerulosclerosis was 93% and for the identification of EXHC was 97%. The pathologists were blinded to the clinical data and renal outcomes.

### Immunohistochemical staining

Sections (3 μm) cut from 10% formalin-fixed, paraffin-embedded kidney specimens were immunostained for complement C9 (ab173302, Abcam, Cambridge, MA, USA) and ApoE (ab183597, Abcam), as previously described^58^. Then the tissues were incubated with an HRP-conjugated goat anti-rabbit antibody (8114, CST, USA). Antibody binding was detected by incubation with a fresh mixture of diaminobenzidine (DAB, 8059S, CST, USA), according to the manufacturer’s instructions. Images were captured with a Nikon DXM 1200/NIS-Elements mounted on a light microscope (Nikon Eclipse E600, Shanghai, CHN) and analysed using Image J software. Complement C9 and ApoE staining in the glomeruli was quantified as ratio of staining-positive area/glomerular tuft area.

### Renal outcomes

The electronic medical records pf the patients were collected and reviewed from the time of renal biopsy to one of the two endpoints: ESRD, or January 2019. Renal survival was the primary end point of the study and was defined by the progression to ESRD, defined by an eGFR < 15 mL/min/1.73 m^2^, or the commencement of renal replacement therapy^11,49^.

### Single glomerular proteomics

Paraffin-embedded specimens were cut into 10-μm thick sections, mounted on Zeiss PEN membrane glass slides, and stained with periodic acid-Schiff. Glomeruli demonstrating solidified glomerulosclerosis or KW nodules were microdissected using a Zeiss dissector (PALM Micro Beam, Oberkochen, Germany) and collected into 500-μL microcentrifuge tube caps. After collecting a total of approximate 100‒120 glomeruli, 40 μL 0.1 M Tris–HCL/dithiothreitol (PH 8.0) was added to the tube. The mixtures were sonicated at a frequency of 40 kHz in an ice-cold bath for 90 min. Then the microdissected fragments were added SDS at a final concentration of 4% with vortexing at 95℃ for 60 min. After cooling to room temperature, the tube was centrifuged at 14,000 × *g* for 10 min, then the supernatant was collected and transferred to a new tube.

Each protein sample was separated by the one-dimensional SDS-PAGE and the corresponding lane was cut into bands for parallel processing. The gel pieces were placed in a digestion solution consisting of 12.5 ng/μL trypsin (Sigma, Proteomics Grade) in 50 mM NH_4_HCO_3_ and vortexed at room temperature until they had absorbed a substantial amount of the solution. The gel pieces were then subjected to enzymatic cleavage at 37℃ overnight, the peptides were extracted, the mixtures were centrifuged, and the supernatants were then transferred to new siliconized tubes. Two extractions were then performed by exchange using 200-μL aliquots of 0.1% formic acid and 50% acetonitrile (ACN) at room temperature for 5 min each. The exchanged fractions were then pooled and dried, whereupon they were ready for LC–MS/MS analysis^59^.

The extracted peptides were subjected to label-free proteomic analysis using a higher-energy collision dissociation (HCD) (Thermo Orbitrap Fusion Lumos Tribrid) and LC–MS/MS. Buffer A consisted of 0.1% (v/v) formic acid (FA) in water and Buffer B consisted of 0.1% (v/v) FA in 80% ACN. The lyophilized peptides were dissolved in Buffer A and were loaded on a self-packed 75-μm-inner-diameter 25-cm-long column (ReproSil-Pur C18-AQ 1.9 μm; Dr. Maisch, Ammerbuch, Germany) for separation over a 78-min gradient at 300 nL/mL. The eluted peptides were introduced directly into the Fusion Lumos. Full-scan MS spectra were obtained using the positive ion mode, with a scanning mass range of mass/charge ratio (m/z) 300‒1,500 and a resolution of 120,000 full width at half maximum (FWHM), full-scan MS spectra were obtained. The HCD MS/MS spectra were then acquired in a data-dependent manner. We selected the most abundant ions in 3 s for HCD fragmentation per MS scan in the Orbitrap at a resolution of 15,000 FWHM. The normalized collision energy was 30% in this procedure.

Peptide identification was performed with at least 99% confidence, as specified by the MaxQuant software (version 1.6.2.6), and a < 1% false discovery rate (FDR), based on forward/reverse human UNIPROT database searches (http://www.uniprot.org, 2018.07). The corresponding proteins identified were subjected to multivariate statistical analysis and functional and/or signalling enrichment analysis as follows. Proteins were considered to have been identified with confidence if at least two unique peptides were detected and if there was an experiment-wide FDR of ≤ 1.0% at both the protein and the peptide levels. For missing data, K-Nearest Neighbour imputation was used to filling the missing data. If a protein was not identified in more than half of the samples, the protein was not included in further analyses. In addition, any protein with a coefficient of variation of > 30% was excluded. The final list of proteins was subjected to further multivariate statistical analysis and functional or/and signalling enrichment analysis.

The proteins underwent relative quantification by normalized using the median centring method and their levels are expressed in a log_2_ normalization format. One-way ANOVA analysis, followed by Benjamini–Hochberg multiple hypothesis correction, was used for statistical comparisons. Proteins with an FDR < 0.05 and a fold change > 1.5 were visualized using a heatmap. In addition, the Wukong Platform was employed for GO^60^, and each protein was subjected to KEGG pathway analysis. Pathways with an FDR threshold of 0.05 were regarded as being significantly different in their representation in the samples. Hierarchical clustering and heat map visualization for the proteins were performed using GraphPad Prism version 8.0^61^.

### Statistical analysis

Continuous variables are presented as means and standard deviations if normally distributed, or as medians and interquartile ranges if not normally distributed. Categorical variables are presented as counts and percentages. Continuous variables were analysed using Student’s *t*-test or the Wilcoxon test, while categorical variables were analysed using the chi-square test or Fisher’s exact test.

Survival curves were generated using Kaplan–Meier method with a log-rank test. Univariate and multivariable Cox proportional hazards models were used to explore the association between clinical or pathological features and ESRD. Baseline 24-h proteinuria and diabetic retinopathy data were missing for 18 and 10 patients, respectively. We first determined if there were any differences in clinical parameters of patients who were or were not missing values, to identify whether these were random omissions. We then used multiple imputation methods to derive multivariable models^12^. The proportional hazards assumption in Cox models was tested to determine whether the datasets satisfied the inherent assumptions of Cox analysis. The Cox proportional hazards model was used to calculate HRs and 95% CIs for progression to ESRD. We used two multivariable Cox proportional hazards models, both of which included clinical parameters (age, sex, ethnicity, systolic blood pressure, diabetic retinopathy, duration of diabetes, baseline serum albumin, haemoglobin, cholesterol, eGFR, proteinuria, haematuria, and use of renin–angiotensin–aldosterone system inhibitors). The first multivariable model was the “Glomerular Model”, which incorporated glomerular parameters that were *P* < 0.1 in univariate models. Glomerular parameters with *P* < 0.1 in the Glomerular Model were then added to the successive multivariable models. Given that several glomerular parameters, such as RPS glomerular class, KW nodules, segmental sclerosis, and mesangial matrix have been reported to be associated with renal outcomes^9,11,13,46^, these parameters were also added to the second multivariable model, regardless of their statistical status. The second multivariable model was the “Fully Adjusted Model”, which incorporated the above glomerular parameters, as well as interstitial and vascular parameters that were *P* < 0.05 in univariate models. Parameters that were *P* < 0.05 in the “Fully Adjusted Model” were considered to be significant prognostic indicators. The incremental prognostic value of including pathological parameters in the model, versus a model that only contained clinical parameters, was analysed by calculating Harrell’s C-statistic and Somer’s D-statistic, calculating the likelihood ratio, and assessing the AIC^62^. Linear regression analyses were used to assess the association between the amount of solidified glomerulosclerosis and the number of KW nodules.

To address death as a competing outcome, the subdistribution hazards model (Fine and Gray method)^14^ was used to explore the association between clinical or pathological features and ESRD. Two multivariable subdistribution hazards models that were similar to the multivariable Cox proportional hazards models were applied in the competing risk analyses.

Statistical analyses were performed using Stata version 14.0 (StataCorp LLC, College Station, TX, USA) or SAS version 9.4 (SAS Institute Inc., Cary, NC, USA). Statistical significance was accepted at *P* < 0.05.

### Ethics approval

All procedures performed in studies involving human participants were in accordance with the ethical standards of the institutional and/or national research committee and with the 1964 Helsinki Declaration and its later amendments or comparable ethical standards. This study was approved by the institutional review board at the West China Hospital of Sichuan University.

## Supplementary Information

Supplementary Information.

## Data Availability

The data that support the findings of this study are available from the corresponding author upon reasonable request.
