# Supplementary material for: Solidified glomerulosclerosis, identified using single glomerular proteomics, predicts end-stage renal disease in Chinese patients with type 2 diabetes
Source: Sci Rep. 2021 Feb 25;11:4658. doi: 10.1038/s41598-021-83856-z (PMC7907371; doi:10.1038/s41598-021-83856-z)
Supplement: Supplementary file 1 — Supplementary Information. [file 41598_2021_83856_MOESM1_ESM.doc]

**Solidified glomerulosclerosis, identified using** **single glomerular proteomics, predicts end-stage renal disease in Chinese patients with type 2 diabetes**

Lijun Zhao^1^, Fang Liu^1*^, Lin Li^2^, Junlin Zhang^1^, Tingli Wang^1^, Rui Zhang^1^, Wei Zhang^3^, Xiaoyan Yang^3^, Xiaoxi Zeng^3^, Yiting Wang^1^, Yucheng Wu^1^, Hao Yang^4,5^, Shisheng Wang^5^, Yi Zhong^5^, Huan Xu^2^, Shanshan Wang^1^, Ruikun Guo^1^, Honghong Ren^1^, Lichuan Yang^1^, Baihai Su^1^, Jie Zhang^4^, Nanwei Tong^6^, Xin J Zhou^7^, Mark E. Cooper^8^

^1^Division of Nephrology, West China Hospital of Sichuan University, Chengdu, Sichuan, China;

^2^Division of Pathology, West China Hospital of Sichuan University, Chengdu, Sichuan, China;

^3^West China Biomedical Big Data Center, West China Hospital/ West China School of Medicine of Sichuan University, Chengdu, Sichuan, China;

^4^Key Laboratory of Transplant Engineering and Immunology, Ministry of Health, Regenerative Medicine Research Center, West China Hospital of Sichuan University, Chengdu, Sichuan, China.

^5^West China-Washington Mitochondria and Metabolism Research Center, West China Hospital of Sichuan University, Chengdu, Sichuan, China;

^6^Division of Endocrinology, West China Hospital of Sichuan University, Chengdu, Sichuan, China;

^7^Department of Pathology, Baylor University Medical Center at Dallas, Dallas, Texas, USA

^8^Department of Diabetes, Central Clinical School, Monash University, Melbourne, Australia

***Corresponding author**: Fang Liu, MD, PhD. Division of Nephrology, West China Hospital of Sichuan University, No. 37, Guoxue Alley, Chengdu, Sichuan Province, China. Telephone: 86-28-18980601214. Fax: 86-28-85422335. Email: [liufangfh@163.com](mailto:liufangfh@163.com)

**Supplementary Table S1. Definitions of pathological findings.**

| Pathological findings | Scores | Definition of score | n (%) |
| --- | --- | --- | --- |
| **Glomerular lesions** |  |  |  |
| RPS glomerular class ^a^ |  |  |  |
|  | I | Mild, nonspecific LM changes with GBM thickening | 17 (5) |
|  | IIa | Mild mesangial expansion in ＞25% of mesangium | 68 (21) |
|  | IIb | Severe mesangial expansion in ＞25% of mesangium | 32 (10) |
|  | III | At least one Kimmelstiel-Wilson nodule | 155 (48) |
|  | IV | Advanced diabetic glomerulosclerosis | 50 (16) |
| Solidified glomerulosclerosis |  | (Number of solidified glomerulosclerosis)/number of all glomeruli (%) |  |
|  | 0 | Absent | 195 (61) |
|  | 1 | 1% -20% | 94 (29) |
|  | 2 | 21% -50% | 33 (10) |
|  | 3 | ＞50% | 0 (0) |
| Ischemia obsolescent glomerulosclerosis |  | (Number of obsolescent glomerulosclerosis)/number of all glomeruli (%) |  |
|  | 0 | Absent | 65 (20) |
|  | 1 | 1% -20% | 112 (35) |
|  | 2 | 21% -50% | 117 (36) |
|  | 3 | ＞50% | 28 (9) |
| Not otherwise specified glomerulosclerosis |  | (Number of not otherwise specified glomerulosclerosis)/number of all glomeruli (%) |  |
|  | 0 | Absent | 270 (84) |
|  | 1 | 1% -20% | 45 (14) |
|  | 2 | 21% -50% | 7 (2) |
|  | 3 | ＞50% | 0 (0) |
| Kimmelstiel-Wilson nodules |  | (Number of glomerulus with KW nodular lesion)/number of all glomeruli (%) |  |
|  | 0 | Absent | 150 (47) |
|  | 1 | 1% -25% | 133 (41) |
|  | 2 | 26% -50% | 34 (11) |
|  | 3 | 51% -75% | 5 (1) |
|  | 4 | ＞75% | 0 (0) |
| Segmental sclerosis |  | (Number of segmental sclerosis)/number of all glomeruli (%) |  |
|  | 0 | Absent | 189 (59) |
|  | 1 | 1% -25% | 119 (37) |
|  | 2 | 26% -50% | 12 (3) |
|  | 3 | 51% -75% | 2 (1) |
|  | 4 | ＞75% | 0 (0) |
| Mesangial expansion |  |  |  |
|  | 1 | Mesangial expansion ＜ Capillary lumen | 64 (20) |
|  | 2 | Mesangial expansion = Capillary lumen | 75 (23) |
|  | 3 | Mesangial expansion ＞ Capillary lumen | 183 (57) |
| Periglomerular fibrosis |  | (Number of periglomerular fibrosis)/number of all glomeruli (%) |  |
|  | 0 | Absent | 78 (24) |
|  | 1 | 1% -25% | 173 (54) |
|  | 2 | 26% -50% | 61 (19) |
|  | 3 | 51% -75% | 7 (2) |
|  | 4 | ＞75% | 3 (1) |
| Glomerular capsular adhesions |  | (Number of capsular adhesion glomerulus)/number of all glomeruli (%) |  |
|  | 0 | Absent | 185 (57) |
|  | 1 | 1% -25% | 117 (36) |
|  | 2 | 26% -50% | 16 (5) |
|  | 3 | 51% -75% | 2 (1) |
|  | 4 | ＞75% | 2 (1) |
| EXHC |  |  |  |
|  | 0 | Absent | 290 (90) |
|  | 1 | Detected one or more lesions in all biopsy specimens | 32 (10) |
| Capillary microaneurysm |  |  |  |
|  | 0 | Absent | 175 (54) |
|  | 1 | Detected one or more lesions in all biopsy specimens | 147 (46) |
| Capsular drop |  |  |  |
|  | 0 | Absent | 298 (93) |
|  | 1 | Detected one or more lesions in all biopsy specimens | 24 (7) |
| Fibrin cap |  |  |  |
|  | 0 | Absent | 274 (85) |
|  | 1 | Detected one or more lesions in all biopsy specimens | 48 (15) |
| Atubular glomeruli |  |  |  |
|  | 0 | Absent | 246 (76) |
|  | 1 | Detected one or more lesions in all biopsy specimens | 76 (24) |
| **Interstitial lesions** |  |  |  |
| IFTA ^a^ |  |  |  |
|  | 0 | No IFTA | 13 (4) |
|  | 1 | 1%- 25% cortex | 172 (54) |
|  | 2 | 25% - 50% cortex | 105 (33) |
|  | 3 | ＞50% cortex | 32 (9) |
| Interstitial inflammation ^a^ |  |  |  |
|  | 0 | Absent | 11 (3) |
|  | 1 | Infiltration only in relation to IFTA | 245 (76) |
|  | 2 | Infiltration in an area without IFTA | 66 (21) |
| Epithelial tubular degeneration |  |  |  |
|  | 1 | Light changes, exhibited a swollen change | 31 (10) |
|  | 2 | Moderate changes, exhibited vacuolar degeneration | 236 (73) |
|  | 3 | Severe changes, exhibited brush border membrane shed and naked | 55 (17) |
| Protein cast |  |  |  |
|  | 0 | Absent | 132 (41) |
|  | 1 | Detected one or more lesions in all biopsy specimens | 190 (59) |
| RBC cast |  |  |  |
|  | 0 | Absent | 299 (93) |
|  | 1 | Detected one or more lesions in all biopsy specimens | 23 (7) |
| **Vascular lesions** |  |  |  |
| Arteriosclerosis ^a^ |  |  |  |
|  | NA | No arteries were present | 4 (1) |
|  | 0 | No intimal thickening | 48 (15) |
|  | 1 | Intimal thickening＜ thickness of media | 154 (48) |
|  | 2 | Intimal thickening≥thickness of media | 116 (36) |
| Arteriolar hyalinosis ^a^ |  |  |  |
|  | NA | No arteriole was present | 11 (3) |
|  | 0 | Absent | 34 (11) |
|  | 1 | At least one area of arteriolar hyalinosis | 84 (26) |
|  | 2 | More than one area of arteriolar hyalinosis | 193 (60) |

^a^ Defined by RPS Diabetic Nephropathy Classification. For Cox proportional hazard analyses, class I and class IIa were collapsed together as the reference group for RPS glomerular class. Scores of 2 and 3 were collapsed together as 2 for solidified glomerulosclerosis, not otherwise specified glomerulosclerosis. Scores of 3 and 4 were collapsed together for Kimmelstiel-Wilson nodules, glomerular capsular adhesions, periglomerular fibrosis. Scores of 2, 3 and 4 were collapsed together for segmental sclerosis. RPS, renal pathology society; DN, diabetic nephropathy; LM, light microscopy; GBM, glomerular basement membrane; KW, Kimmelstiel–Wilson; EXHC, extra-capillary hypercellularity; RBC, red blood cell; IFTA, Interstitial fibrosis and tubular atrophy.

**Supplementary Table S2. Pathological characteristics of patients with or without end-stage renal disease**

|  | ESRD (+) | ESRD (–) | *P* value |
| --- | --- | --- | --- |
|  | (n = 144) | (n = 178) |  |
| **Glomerular lesions** |  |  |  |
| RPS classification ^a^, n (%) |  |  | <0.001 |
| I | 0 (0) | 17 (9) |  |
| IIa | 10 (7) | 58 (33) |  |
| IIb | 10 (7) | 22 (12) |  |
| III | 99 (69) | 56 (32) |  |
| IV | 25 (17) | 25 (14) |  |
| % Global glomerulosclerosis ^b^, median (IQR) | 30.0 (16.0–50.0) | 20.0 (8.3–44.4) | 0.01 |
| - % Solidifed GS ^b^, median (IQR) | 4.6 (0–15.2) | 0 (0–3.9) | <0.001 |
| - % Ischemia obsolescent GS ^b^, median (IQR) | 20.0 (9.2–33.3) | 16.7 (4.2–33.3) | 0.33 |
| - % Not otherwise specified GS ^b^, median (IQR) | 0 (0–0) | 0 (0–0) | 0.20 |
| % KW ^b^, median (IQR) | 12.5 (4.0–20.0) | 0 (0–9.5) | <0.001 |
| % Segmental Sclerosis ^b^, median (IQR) | 0 (0–12.5) | 0 (0–9.1) | 0.13 |
| % Periglomerular fibrosis ^b^, median (IQR) | 14.3 (8.3–25.0) | 11.1 (0–22.2) | 0.03 |
| % Glomerular capsular adhesions ^b^, median (IQR) | 5.0 (0–13.8) | 0 (0–9.1) | 0.002 |
| Mesangial expansion, median (IQR) | 3 (2–3) | 2 (1–3) | <0.001 |
| EXHC, n (%) | 25 (17) | 7 (4) | 0.02 |
| Capillary microaneurysm, n (%) | 88 (61) | 59 (33) | <0.001 |
| Capsular drop, n (%) | 16 (11) | 8 (5) | 0.02 |
| Fibrin cap, n (%) | 20 (14) | 28 (16) | 0.65 |
| Atubular glomeruli, n (%) | 46 (32) | 30 (17) | 0.002 |
| **Interstitial lesions** |  |  |  |
| IFTA, median (IQR) | 2 (1–2) | 1 (1–2) | <0.001 |
| Tubular epithelial degeneration, median (IQR) | 2 (2–2) | 2 (2–2) | 0.02 |
| Protein cast, n (%) | 87 (60) | 103 (94) | 0.64 |
| RBC cast, n (%) | 13 (9) | 10 (6) | 0.24 |
| Interstitial inflammation, median (IQR) | 1 (1–2) | 1 (1–1) | <0.001 |
|  |  |  |  |
| **Vascular lesions** |  |  |  |
| Arteriosclerosis, median (IQR) | 1 (1–2) | 1 (1–2) | 0.24 |
| Arteriolar hyalinosis, median (IQR) | 2 (1–2) | 2 (1–2) | 0.10 |

Data are shown as median (25^th^-75^th^ percentiles) or as number (percentage) of patients.

^a^ Defined by RPS Diabetic Nephropathy Classification.

^b^ Glomerular lesions were quantified as the percentage of the total number of glomeruli for each biopsy.

Mesangial expansion score (1 to 3 scale); Tubular epithelial degeneration score (1 to 3 scale); Interstitial inflammation (0 to 2 scale); Arteriolar hyalinosis (0 to 2 scale); Arteriosclerosis (0 to 2 scale) were shown as median score (25^th^– 75^th^ percentiles).

IQR, interquartile range; RPS, Renal Pathology Society; SolGS, solidified glomerulosclerosis; ObsGS, ischemia obsolescent glomerulosclerosis; NOSGS, not otherwise specified glomerulosclerosis; KW, Kimmelstiel–Wilson; SS, Segmental Sclerosis; EXHC, extra-capillary hypercellularity; IFTA, interstitial fibrosis and tubular atrophy; RBC, red blood cell.

**Supplementary Table S3. Pathological predictors of end-stage renal disease**

| Statistics | Model 1 ^a^ | Model 1 + solidified GS | Model 1 + EXHC |
| --- | --- | --- | --- |
| Harrell's C–statistics | 0.794 | 0.799* | 0.800* |
| Somers' D–statistics | 0.589 | 0.596 | 0.600 |
| LR χ^2^ | 132.6 | 140.2* | 138.2* |
| AIC | 1301.3 | 1295.7 | 1296.7 |

^a^ Model 1 included serum albumin, hemoglobin, duration of diabetes, estimated glomerular filtration rate, and urinary protein. SolGS, solidified glomerulosclerosis; EXHC, extra-capillary hypercellularity; LR χ^2^, likelihood ratio Chi–square statistics; AIC, Akaike information criterion. *, P＜0.05 (versus Model 1).

**Supplementary Table S4. Adjusted hazard ratios for predicting progression to end-stage renal disease in patients with follow up at least 1 year, 2 years or 3 years.**

|  | Patients followed up≥1 year | |  | Patients followed up≥ 2 years | |  | Patients followed up ≥3 years | |
| --- | --- | --- | --- | --- | --- | --- | --- | --- |
| Characteristics | Adjusted HR^a^ (95% CI) | *P* value |  | Adjusted HR^a^ (95% CI) | *P* value |  | Adjusted HR^a^ (95% CI) | *P* value |
| Solidified GS |  |  |  |  |  |  |  |  |
| 0 | 1 (reference) |  |  | 1 (reference) |  |  | 1 (reference) |  |
| 1 | 1.80 (1.08–2.96) | 0.03 |  | 1.84 (1.13–3.02) | 0.02 |  | 2.05 (1.19–3.54) | 0.01 |
| 2+3 | 2.53 (1.51–4.92) | <0.01 |  | 2.94 (1.58–5.49) | <0.01 |  | 2.37 (1.17–4.81) | 0.02 |
| Presence of EXHC | 2.78 (1.59–5.17) | <0.001 |  | 2.93 (1.61–5.34) | <0.001 |  | 2.77 (1.38–5.55) | <0.01 |

^a^ Adjusting for age, sex, ethnicity, systolic blood pressure, duration of diabetes, diabetic retinopathy, haematuria, serum albumin, haemoglobin, cholesterol, eGFR, proteinuria at the time of biopsy, renin-angiotensin-aldosterone system inhibitor use, the calendar years of recruitment, and pathological parameters that with *P*<0.1 in the “Glomerular Model”. HR, hazard ratio; CI, confidence interval; GS, glomerulosclerosis; EXHC, extra-capillary hypercellularity.

**Supplementary Table S5. The 5-year renal survival rates according to different pathological changes**

| Pathological findings |  | 5-year renal survival | *P* value |
| --- | --- | --- | --- |
| RPS glomerular class |  |  | <0.001 |
|  | Class I+IIa | 78% |  |
|  | Class IIb | 53% |  |
|  | Class III | 15% |  |
|  | Class IV | 19% |  |
| IFTA |  |  | <0.001 |
|  | Score 0 | 74% |  |
|  | Score 1 | 41% |  |
|  | Score 2+3 | 21% |  |
| Solidified GS |  |  | <0.001 |
|  | Score 0 | 50% |  |
|  | Score 1 | 18% |  |
|  | Score 2 | 1% |  |
| EXHC |  |  | <0.001 |
|  | Absent | 41% |  |
|  | Detected | 13% |  |

RPS, Renal Pathology Society; IFTA, interstitial fibrosis and tubular atrophy; SolGS, solidified glomerulosclerosis; EXHC, extra-capillary hypercellularity.

**Supplementary Table S6. Clinical characteristics of patients stratified by solidified glomerulosclerosis .**

| Characteristics | SolGS (+) | SolGS (–) | *P* value |
| --- | --- | --- | --- |
|  | (n = 127) | (n = 195) |  |
| Age, mean (SD), y | 51 (9) | 52 (10) | 0.52 |
| Sex, Male, n (%) | 78 (61) | 144 (74) | 0.02 |
| Ethnicity |  |  | 0.10 |
| Han, n (%) | 119 (94) | 172 (88) |  |
| Tibetan, n (%) | 8 (6) | 23 (12) |  |
| Smoking, Never/Ex/Current, n | 76/20/31 | 101/30/64 | 0.25 |
| History of Hypertension, n (%) | 111 (87) | 162 (83) | 0.29 |
| BMI, mean (SD), Kg/m^2^ | 25.6 (4.1) | 25.4 (3.5) | 0.68 |
| Duration of diabetes, median (IQR), months | 84 (36–132) | 84 (36–132) | 0.66 |
| History of DR, n (%) | 70 (56) | 77 (41) | 0.01 |
| HbA1c, median (IQR), % | 6.7 (5.9–8.1) | 7.1 (5.9–8.3) | 0.36 |
| FPG, median (IQR), mg/dL | 131.4 (99.0–174.6) | 129.6 (102.6–169.2) | 0.98 |
| Haemoglobin, mean (SD), g/L | 111.2 (22.7) | 125.0 (29.1) | <0.001 |
| Serum albumin, mean (SD), g/L | 31.3 (7.5) | 35.2 (7.8) | <0.001 |
| 24–h proteinuria, median (IQR), g/d | 6.0 (3.5–9.1) | 3.1 (1.3–5.8) | <0.001 |
| Hematuria, n (%) | 57 (45) | 79 (41) | 0.44 |
| UA, mean (SD), mg/dL | 6.6 (1.5) | 6.5 (1.3) | 0.45 |
| Triglyceride, median (IQR), mg/dL | 159.5 (124.0–212.6) | 150.6 (106.3–203.8) | 0.09 |
| Cholesterol, median (IQR), mg/dL | 201.0 (174.0–247.4) | 185.6 (154.6–220.4) | 0.01 |
| HDL, median (IQR), mg/dL | 50.3 (42.5–61.9) | 50.3 (38.7–61.9) | 0.27 |
| LDL, median (IQR), mg/dL | 116.0 (96.7–154.6) | 104.4 (81.2–139.2) | 0.01 |

Data are presented as the mean (standard deviation) for continuous variables with symmetric distribution, median (25^th^–75^th^ percentiles) for continuous variables with asymmetric distribution, or percentages for categorical variables. SD, standard deviation; IQR, interquartile range; BMI, body mass index; DR, diabetic retinopathy; FPG, fasting plasma glucose; UA, uric acid; HDL, high–density lipoprotein cholesterol; LDL, low–density lipoprotein cholesterol; SolGS, solidified glomerulosclerosis.

**Supplementary Table S7. The proteins identified by laser microdissection and liquid chromatography electrospray tandem mass spectrometry in solidified glomerulosclerosis and Kimmelstiel-Wilson nodule.**

| Protein ID | Protein name | log2  Fold Change | Threshold |  | Protein ID | Protein name | log2 Fold Change | Threshold |
| --- | --- | --- | --- | --- | --- | --- | --- | --- |
| P01024 | Complement C3 | 0.9144 | UP |  | P07355 | Annexin A2 | 0.0068 | NoChange |
| P02649 | Apolipoprotein E | 1.4269 | UP |  | P08670 | Vimentin | -0.5524 | NoChange |
| P02743 | Serum amyloid P-component | 0.8158 | UP |  | P0DOX5 | Immunoglobulin gamma-1 heavy chain | 0.4868 | NoChange |
| P02748 | Complement component C9 | 1.4506 | UP |  | P0DOX6 | Immunoglobulin mu heavy chain | 0.5288 | NoChange |
| P06576 | ATP synthase subunit beta, mitochondrial | 1.5048 | UP |  | P12110 | Collagen alpha-2(VI) chain；COL6A2 | 0.3692 | NoChange |
| P07360 | Complement component C8 gamma chain | 1.3796 | UP |  | P12111 | Collagen alpha-3(VI) chain；COL6A3 | 0.4724 | NoChange |
| P08572 | Collagen alpha-2(IV) chain | 0.8076 | UP |  | P14625 | Endoplasmin | -1.5678 | NoChange |
| P14618 | Pyruvate kinase PKM | 0.6126 | UP |  | P15924 | Desmoplakin | 0.2880 | NoChange |
| P22626 | Heterogeneous nuclear ribonucleoproteins A2/B1 | 0.7400 | UP |  | P16403 | Histone H1.2 | 0.0464 | NoChange |
| P24821 | Tenascin | 1.1183 | UP |  | P18206 | Vinculin | 0.0807 | NoChange |
| P68871 | Hemoglobin subunit beta | 0.6318 | UP |  | P21333 | Filamin-A | 0.3298 | NoChange |
| P04083 | Annexin A1 | -0.8260 | DOWN |  | P25705 | ATP synthase subunit alpha, mitochondrial | 0.3935 | NoChange |
| P11142 | Heat shock cognate 71 kDa protein | -1.2918 | DOWN |  | P55268 | Laminin subunit beta-2 | 0.3787 | NoChange |
| P35579 | Myosin-9 | -1.1421 | DOWN |  | P60174 | Triosephosphate isomerase | 0.1413 | NoChange |
| P48681 | Nestin | -0.8837 | DOWN |  | P62805 | Histone H4 | 0.1960 | NoChange |
| P62834 | Ras-related protein Rap-1A | -1.1421 | DOWN |  | P63261 | Actin, cytoplasmic 2；ATG1 | -0.1146 | NoChange |
| Q96JY6 | PDZ and LIM domain protein 2 | -1.1537 | DOWN |  | P81605 | Dermcidin | -0.2175 | NoChange |
| O15230 | Laminin subunit alpha-5 | 0.4401 | NoChange |  | P98160 | Basement membrane-specific heparan sulfate proteoglycan core protein | 0.0787 | NoChange |
| P01876 | Immunoglobulin heavy constant alpha 1 | -0.4204 | NoChange |  | Q01995 | Transgelin | 0.3080 | NoChange |
| P02462 | Collagen alpha-1(IV) chain | 0.3027 | NoChange |  | Q02413 | Desmoglein-1 | 0.4201 | NoChange |
| P02545 | Prelamin-A/C | -0.1327 | NoChange |  | Q5VTE0 | Putative elongation factor 1-alpha-like 3 | 0.2975 | NoChange |
| P02671 | Fibrinogen alpha chain | -0.1997 | NoChange |  | Q71DI3 | Histone H3.2 | -0.2585 | NoChange |
| P02751 | Fibronectin | -0.1014 | NoChange |  | Q99878 | Histone H2A type 1-J | 0.0289 | NoChange |
| P04075 | Fructose-bisphosphate aldolase A | 0.4943 | NoChange |  | Q99879 | Histone H2B type 1-M | 0.3158 | NoChange |
| P04406 | Glyceraldehyde-3-phosphate dehydrogenase；GAPDHd | -0.1187 | NoChange |  | Q9BQE3 | Tubulin alpha-1C chain | 0.2327 | NoChange |
| P05062 | Fructose-bisphosphate aldolase B | -0.7474 | NoChange |  | Q9Y490 | Talin-1 | -0.0612 | NoChange |
| P05089 | Arginase-1 | -2.8571 | NoChange |  |  |  |  |  |

**
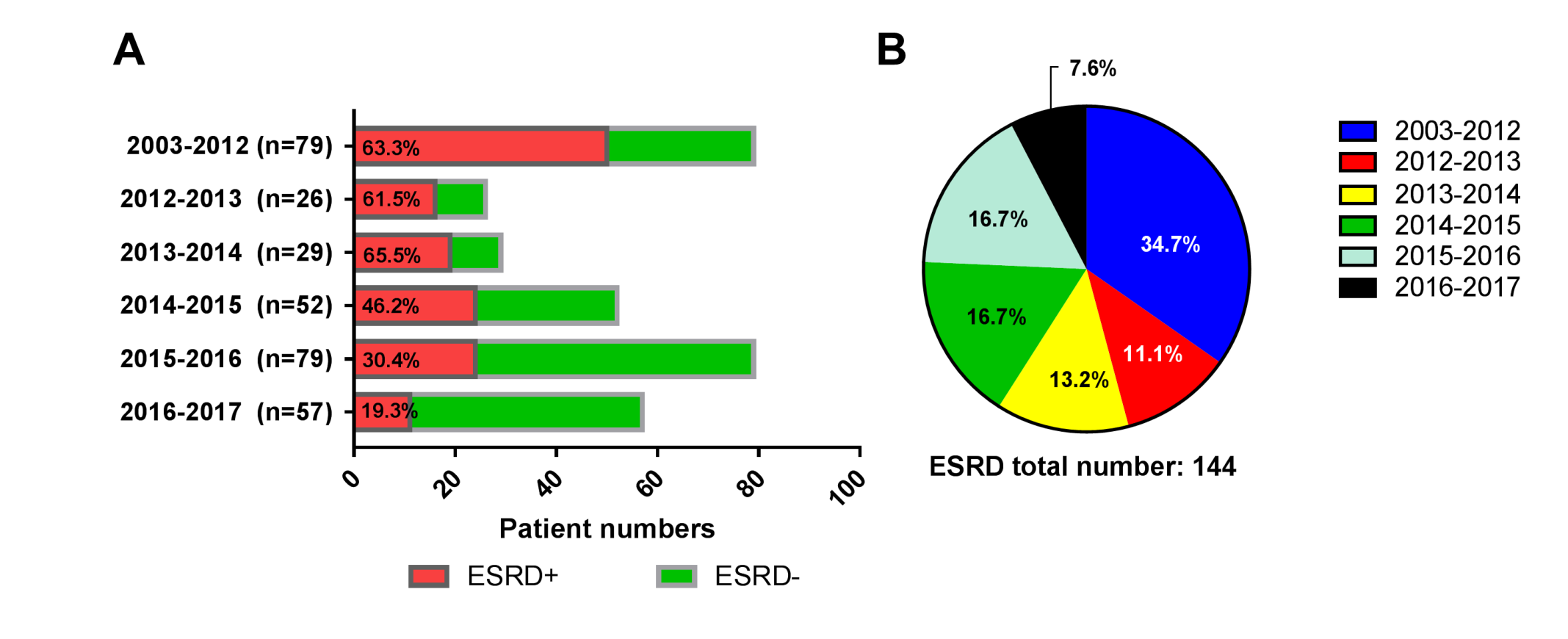
**

**Supplementary Figure S1. The number of patients enrolled in different years.** The red pillars were patients progressed to end-stage renal disease (ESRD) at the end of the study. Red：progressed to ESRD, green：non progressed to ESRD. Numbers in red squares expressed the percentage of patients with ESRD in the specified year.


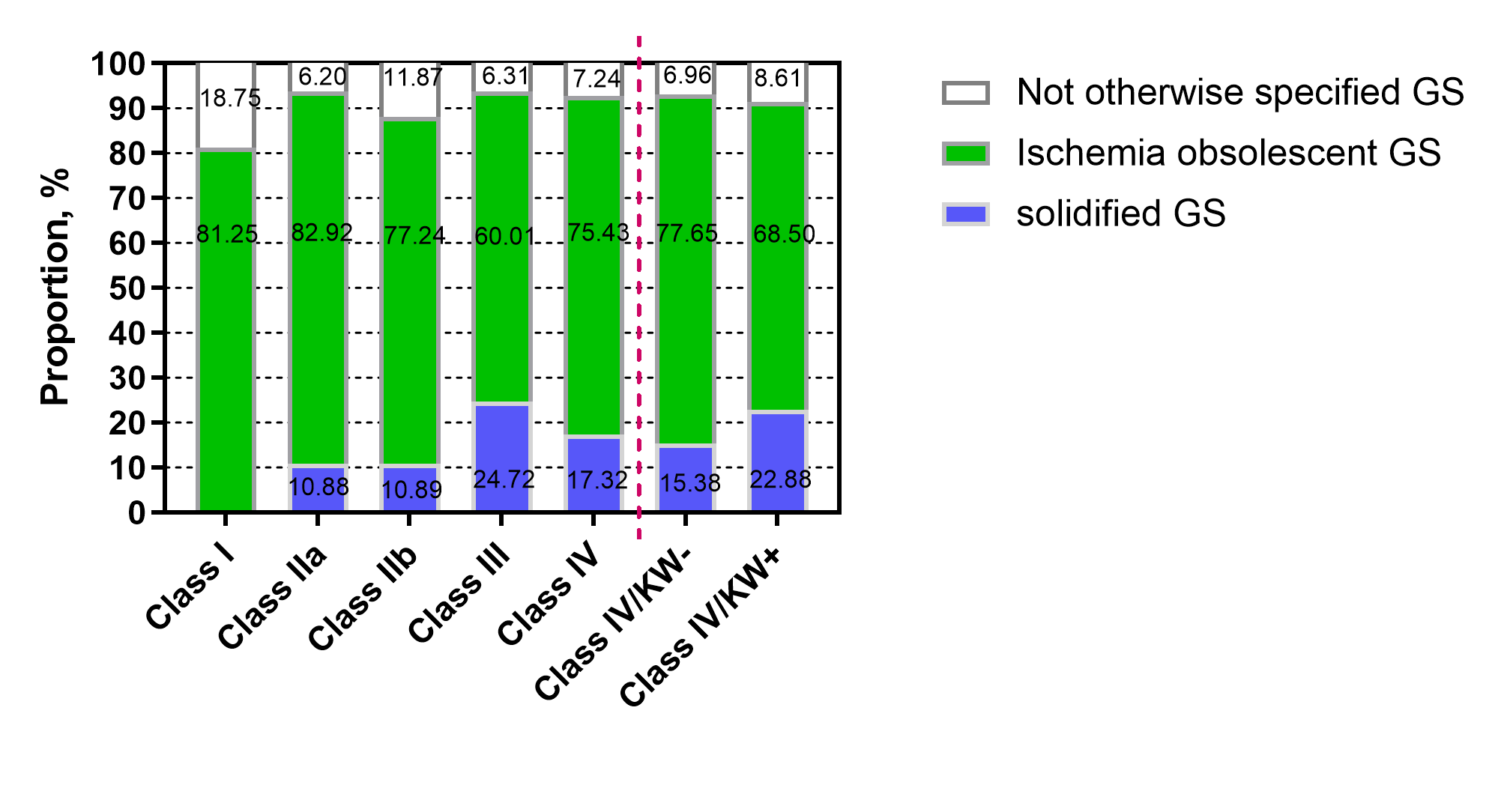
**Supplementary Figure S2. Proportions of each global glomerulosclerosis category in all global glomerulosclerosis stratified by different Renal Pathology Society glomerular classes.** Blue squares=Proportion of solidified glomerulosclerosis (GS), Green squares = Proportion of ischemia obsolescent GS, White squares= Proportion of not otherwise specified GS. Proportion numbers were illustrated in each square.


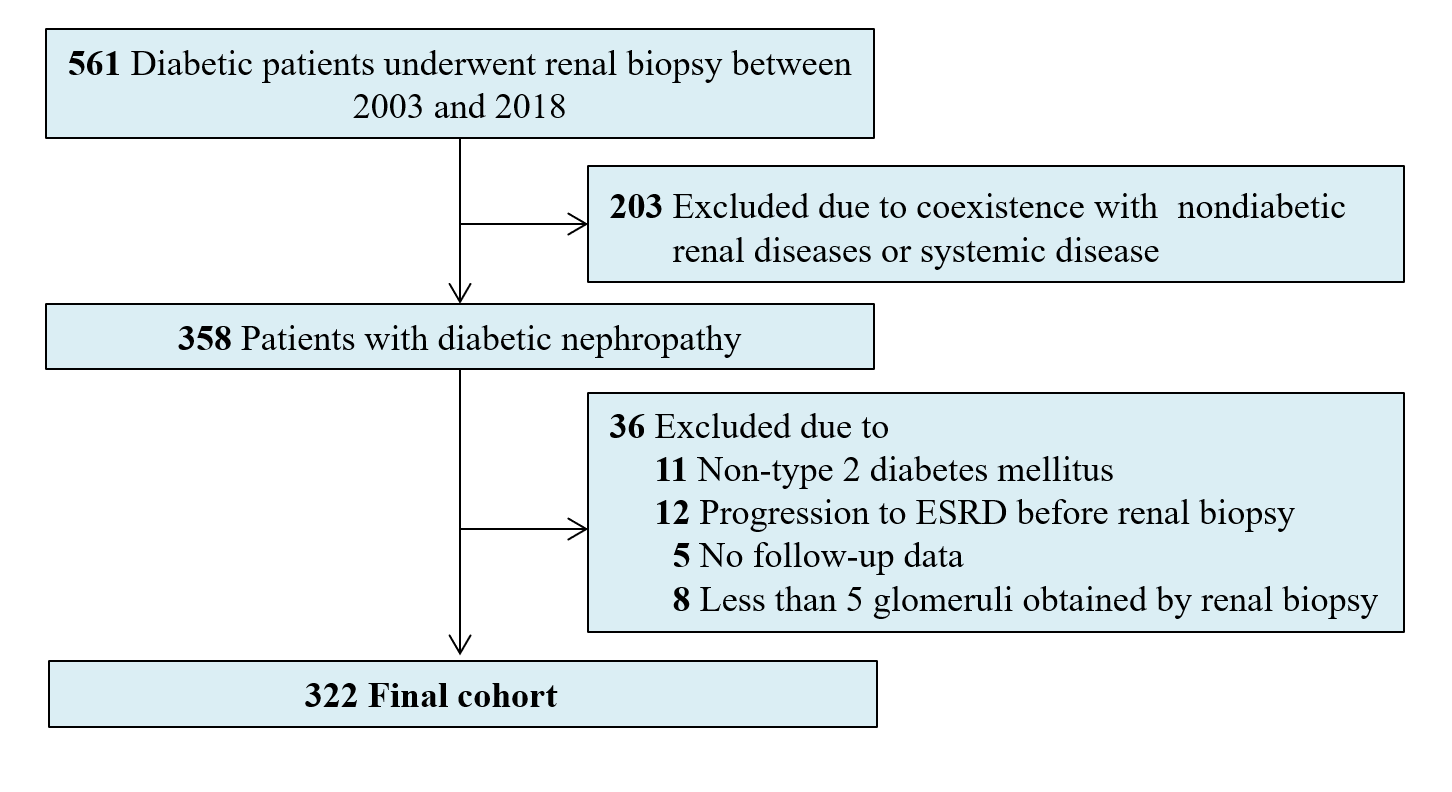


**Supplementary Figure S3. Flowchart of the study participants.**

**
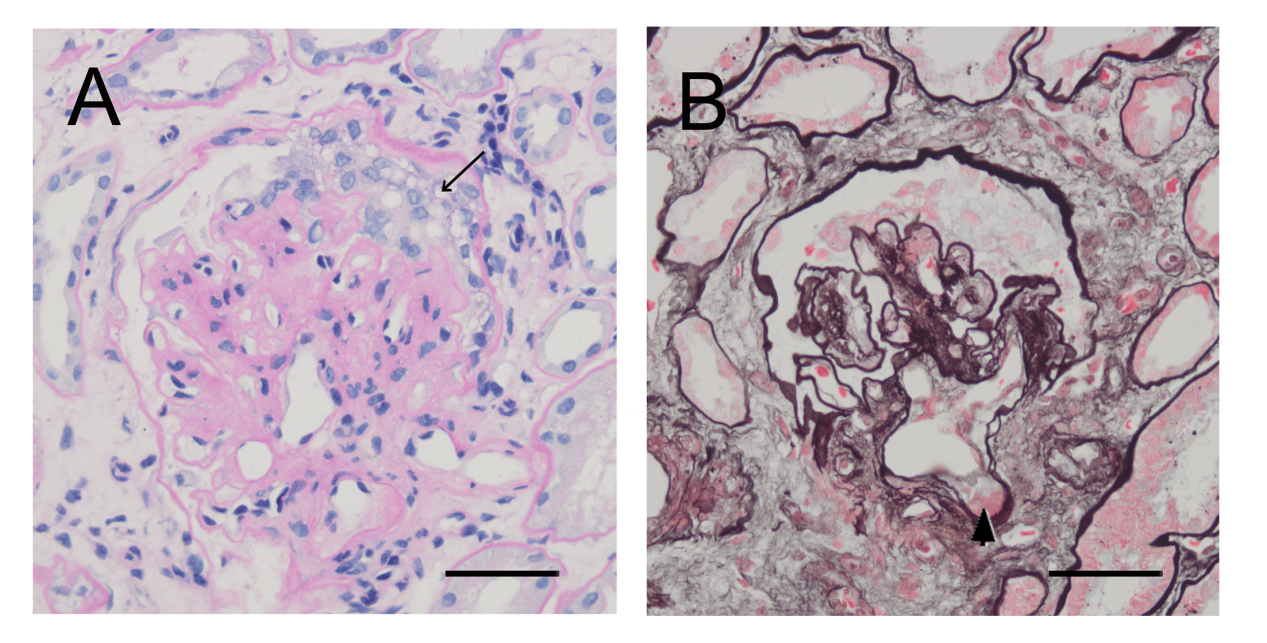
 Supplementary Figure S4. Light micrograph of extra**–**capillary hypercellularity in diabetic nephropathy.** (A) Extra–capillary hypercellularity shows more than two layers of cells (arrows) are aligned around the glomerular tuft in the Bowman’s space (PAS, ×400). (B) Next section of the same glomerulus in panel A. Artery hyalinosis (arrowhead) is presented in the methenamine silver stains (PASM, ×400). Bar= 50 µm.
